# Discrepancies in digital hematopathology diagnoses for consultation and expert panel analysis

**DOI:** 10.1007/s00428-020-02907-4

**Published:** 2020-08-25

**Authors:** Michiel van den Brand, Peet T. G. A. Nooijen, Kimberly D. van der Laan, Peter C. de Bruin, A. M. (Gijs) van Leeuwen, Jan Willem Leeuwis, Jos W. Meijer, Irene Otte-Höller, Konnie M. Hebeda

**Affiliations:** 1 Department of Pathology, Radboud University Medical Center, P. O. Box 9101, 6500 HB Nijmegen, The Netherlands; 2 Pathology-DNA, Rijnstate Hospital, Arnhem, The Netherlands; 3 Pathology-DNA, Jeroen Bosch Hospital, Den Bosch, The Netherlands; 4 Pathology-DNA, St. Antonius Hospital, Nieuwegein, The Netherlands

**Keywords:** Digital pathology, Hematopathology, Lymphoma, Consultation

## Abstract

Digital pathology with whole-slide imaging (WSI) has a large potential to make the process of expert consultation and expert panel diagnosis more rapid and more efficient. However, comparison with the current methods is necessary for validation of the technique. In this study, we determined if digital assessment of whole-slide images of hematopathology specimens with a focus on the assessment of lymphoma can be used for consultation and panel diagnostics. Ninety-three histological specimens with a suspicion for lymphoma were assessed both with conventional microscopy and digital microscopy with a wash out period between assessments. A consensus diagnosis was based on full concordance between the pathologists or, in case of discordances, was reached at a joint session at a multi-headed microscope. In 81% of the cases, there was a full concordance between digital and light microscopical assessment for all three pathologists. Discordances between conventional microscopy and digital pathology were present in 3% of assessments. In comparison with the consensus diagnosis, discordant diagnoses were made in 5 cases with digital microscopy and in 3 cases with light microscopy. The reported level of confidence and need for additional investigations were similar between assessment by conventional and by digital microscopy. In conclusion, the performance of assessment by digital pathology is in general comparable with that of conventional light microscopy and pathologists feel confident using digital pathology for this subspecialty.

## Introduction

A correct histological diagnosis of malignant lymphoma is essential for adequate treatment and accurate prognostication. However, hematopathological diagnosis of lymphoma is known for significant inter- and intra-observer variability. The percentage of discordance between pathologists in the World Health Organization classification from 2008 ranges from 6 to 27% between studies, depending on the case mix [1–3]. Expert panels can help to decrease inter-observer variation. Indeed, the introduction of a panel of expert hematopathologist in the East Netherlands in 2000 resulted in a decrease in discordance from 14 to 9% in a period of 5 years [2].

Although expert panels have proved to be an excellent instrument to improve the quality of hematopathological diagnostics, it does come with practical disadvantages. First of all, the panel members generally do not meet daily, causing a delay in the panel diagnosis. Also, especially in panels that cover large geographical regions, travelling time can be an issue. The current development and implementation of digital pathology and whole-slide imaging (WSI) in the Netherlands provided the opportunity to, partially or completely, perform expert panel diagnostics digitally, thereby shortening the time to expert diagnosis and reducing travelling time. A nationwide digital network PIE (Pathology Image Exchange) was implemented in 2018 and will allow access to digital consultation for all Dutch pathology departments [4].

Although a multitude of studies has been performed to validate whole-slide imaging, these studies mostly focused on surgical pathology specimens with only a limited number of lymph node samples [5, 6]. Recently, one study reported the application of whole-slide imaging to lymphoma diagnosis with a high concordance rate, but there was only a single pathologist who performed the assessments in this study [7].

In the study presented here, we determined if digital assessment of whole-slide images of hematopathology specimens with a focus on the assessment of lymphoma can be used for consultation and panel diagnostics.

## Materials and methods

### Case selection

Ninety-three sequential histological samples with a clinical suspicion for lymphoma were selected from the routine diagnostic service at the Department of Pathology, Rijnstate Hospital, Arnhem, the Netherlands. Bone marrow biopsies were excluded. The primary diagnosis was made between August 2016 and January 2017. The 2 pathologists from other hospitals were already used to reviewing hematopathology slides of this hospital. Pathologists were provided with anonymized patient information including gender, age, clinical question, and relevant history. The diagnostic categories included reactive conditions (*n* = 12), small B cell lymphoma (*n* = 32), large B cell lymphoma (*n* = 27), Hodgkin lymphoma (*n* = 14), T cell lymphoma (*n* = 5), cutaneous lymphoma (*n* = 2), and one case of suspected Langerhans cell histiocytosis. This study was performed according to local and national ethical guidelines and the Declaration of Helsinki.

### Acquisition and assessment of digital slides

Slides were scanned at × 40 equivalent with a Philips IntelliSite Ultra Fast Scanner (Philips Digital Pathology, Best, the Netherlands). Images were first assessed by three hematopathologists (KH, PN, MB) with the Philips Image Management System on a regular desktop computer screen, resulting in the digital microscopy diagnosis (DMD). There was no standard setup used for digital pathology; the pathologist assessed the cases on their regular computer equipment (i.e., standard desktop setup with normal mouse). All pathologists did not yet use digital pathology on a daily basis during the time of the study. After a wash out period of at least 3 months, the same cases were assessed again by the same pathologists with a regular light microscope, resulting in the conventional microscopy diagnosis (CMD). Light microscopical examination was performed with the exact same clinical information and the same slides. If additional information was available or additional stainings had been performed between digital and light microscopical assessment, these were not included.

**Fig. 1:**
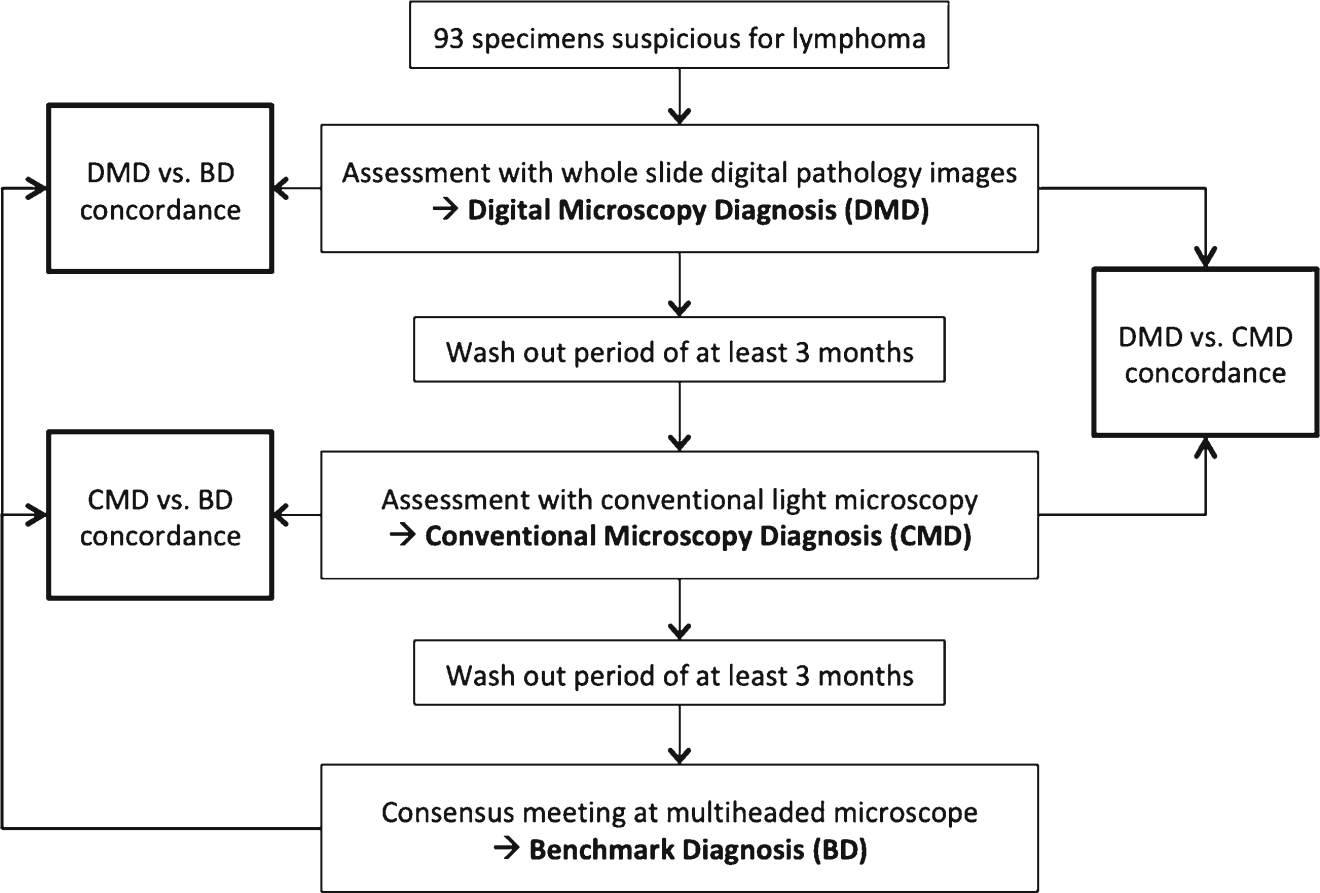
Flow of the study, showing the timing of the different assessments and evaluation of the different levels of concordance

The benchmark diagnosis (BD) was based on light microscopical assessment by all three pathologists. In discordant cases, the BD was established during a discussion by all three pathologists using a multi-headed microscope, again after a wash out period of at least 3 months and without knowing the previous results. The flow of the study is summarized in Fig. 1.

Both for digital and light microscopical assessments, each pathologist was asked to score the following items: diagnosis; level of confidence (very unsure, unsure, sure, very sure); need for additional immunohistochemical stains or molecular analyses (yes/ no); which additional investigations; and any encountered problems during the assessment.

The results were entered into a database, and the assessment of digital and glass slides was assessed for concordance. Major discordances were defined as differences in diagnosis with an impact on treatment, e.g., reactive vs. malignant, a significant change of diagnosis, and presence or absence of transformation. Follicular lymphoma grades 1–3A vs. follicular lymphoma grade 3B was considered discordant. Follicular lymphoma grade 3B vs. diffuse large B cell lymphoma was not considered a major discordance.

Statistical analyses were performed with IBM SPSS statistics for Macintosh, version 26.0 (IBM Corp, Armonk, NY).

## Results

### Concordance between digital microscopy diagnosis and conventional microscopy diagnosis

Three pathologists assessed 93 cases both by digital and light microscopical assessment. In 75 cases (81%), there was a full concordance between DMD and LMD for all three pathologists (Table 1). Major intra-observer discordances for at least one of the pathologists between DMD and LMD were present in 8 cases (9%), with only one case with a discordant assessment by two pathologists (9 discordant assessments out of 279, 3%). Minor intra-observer discordances were present in 10 cases (11%) with none of these cases showing discordance for more than one pathologist. Minor discordances mostly consisted of a less certain diagnosis with a request for additional investigations (*n* = 9) and one case of grade 3B follicular lymphoma vs. diffuse large B cell lymphoma. Analysis of intra-observer agreement including both major and minor discrepancies between DMD and LMD showed Cohen’s kappa values of 0.925, 0.862, and 0.924 for pathologists 1, 2, and 3, respectively.

**Table 1:** Overview of the results

Consensus diagnosis	DMD vs. BD, cases with major discordance*	CMD vs. BD, cases with major discordance*	DMD vs. CMD, cases with major discordance*
Reactive (*n* = 12)	0 (0%)	1 (8%)	1 (8%)
Small B cell lymphoma (*n* = 32) • Follicular lymphoma (*n* = 14) • Marginal zone lymphoma (*n* = 7) • Chronic lymphocytic leukemia/small lymphocytic lymphoma (*n* = 3) • Mantle cell lymphoma (*n* = 4) • Low-grade B cell lymphoma, NOS (*n* = 4, one with suspicion of transformation)	4 (13%)	1 (3%)	5 (16%)
Large B cell lymphoma (*n* = 27) • Diffuse large B cell lymphoma (*n* = 26) • Follicular lymphoma grade 3B with transformation (*n* = 1)	0 (0%)	1 (4%)	1 (4%)
Hodgkin lymphoma (*n* = 14) • Classical Hodgkin lymphoma (*n* = 12) • Nodular lymphocyte predominant Hodgkin lymphoma (*n* = 2)	1 (7%)	0 (0%)	1 (7%)
T cell lymphoma (*n* = 5) • Peripheral T cell lymphoma, NOS (*n* = 2) • Anaplastic large cell lymphoma, ALK+ (*n* = 1) • Anaplastic large cell lymphoma, ALK− (*n* = 1) • Angio-immunoblastic T cell lymphoma (*n* = 1)	0 (0%)	0 (0%)	0 (0%)
Other (*n* = 3) • Cutaneous lymphoma (primary cutaneous follicle center lymphoma, *n* = 2) • Langerhans cell histiocytosis (*n* = 1)	0 (0%)	0 (0%)	0 (0%)
Total (*n* = 93)	5 (5%)	3 (3%)	8 (9%)

### Concordance between the benchmark diagnosis and DMD or CMD

A discordance between the BD and DMD by at least one of the pathologists was present in five cases (5%). In three of these cases (cases 087, 088, and 093), the BD was a low-grade B cell lymphoma, but with digital pathology, a diagnosis of transformation to aggressive lymphoma was made. In another case with discordance (case 082), the BD was classical Hodgkin lymphoma but with digital pathology a diagnosis of T cell lymphoma was made. In the final case with discordance (case 055), the BD was follicular lymphoma with an atypical Hodgkin-like proliferation, but with digital pathology, a diagnosis of classical Hodgkin lymphoma was made.

A discordance between the BD and CMD by at least one of the pathologists was present in three cases (3%). In one case (case 088), the BD was low-grade lymphoma, but transformation was diagnosed by one of the pathologists by conventional microscopy. In the second case (case 006), the BD was that of a reactive condition, but one of the pathologists made a diagnosis of a low-grade B cell lymphoma by conventional microscopy. In the final case (case 037), the BD was a follicular lymphoma grade 3B in transformation vs. a conventional microscopy diagnosis of florid follicular hyperplasia (Table 2).

**Table 2:** Cases with discordances in assessment

Case no.	Gender	Age (year)	Localization and type of material	Type of discordance^a^	Benchmark diagnosis	Path #1 DMD	Path #1 CMD	Path #2 DMD	Path #2 CMD	Path #3 DMD	Path #3 CMD	Comment
006	Male	55	Lung NCB	BD vs. CMD DMD vs. CMD	Reactive	**Reactive**	***LGBCL, NOS***	*Reactive*	Reactive	Reactive	Reactive	One pathologist favoring LGBCL on CMD
055	Male	59	Lymph node incision biopsy	BD vs. DMD DMD vs. CMD	FL (with Hodgkin-like proliferation)	***cHL***	**FL**	FL	FL	FL	FL	One pathologist not recognizing FL component in DMD
037	Female	73	Lymph node excision	BD vs. CMD DMD vs. CMD	FL grade 3B with transformation	**DLBCL**	***Reactive***	FL grade 3B	DLBCL	FL grade 3B	FL grade 3B	One pathologist considering florid hyperplasia on CMD
076	Female	58	Lymph node incision biopsy	DMD vs. CMD	LGBCL, NOS with suspicion for transformation	**MZL**	**DLBCL**	FL	MZL	**LGBCL, NOS**	**DLBCL**	Difficult case, discussion on absence or presence of transformation
082	Female	37	Lymph node NCB	BD vs. DMD DMD vs. CMD	*cHL*	***T-cell lymphoma***	**cHL**	cHL	cHL	cHL	cHL	Needle biopsy. One pathologist with a diagnosis of ALCL on DMD.
087	Male	50	Lymph node incision biopsy	BD vs. DMD DMD vs. CMD	FL	FL	FL	***DLBCL***	**FL**	FL	FL	One pathologist with a diagnosis of FL with transformation on DMD
088	Female	61	Nasopharynx biopsy	BD vs. DMD BD vs. CMD DMD vs. CMD	*MZL*	*DLBCL*	*DLBCL*	***DLBCL***	**MZL**	MZL	MZL	Comment on difficulty to assess nuclei with digital pathology
093	Male	65	Stomach biopsy	BD vs. DMD DMD vs. CMD	MZL	Suspicious for lymphoma	MZL	***DLBCL***	**MZL**	MZL	MZL	Comment on difficulty to assess nuclear size with digital patholgy

Discordances with the BD are shown in italic, discordances between the DMD and CMD are shown in bold.

*ALCL*, anaplastic large cell lymphoma; *BD*, benchmark diagnosis; *cHL*, classical Hodgkin lymphoma; *CMD*, conventional microscopy diagnosis; *DLBCL*, diffuse large B cell lymphoma; *DMD*, digital microscopy diagnosis; *FL*, follicular lymphoma; *LGBCL*, low-grade B cell lymphoma; *MZL*, marginal zone lymphoma; *NCB*, needle core biopsy; *Path*, pathologist

^a^In the scoring by the pathologists, discordances between DMD and CMD are indicated with a gray fill

### Level of confidence and the need for additional investigations

The average level of confidence of the diagnosis was similar between digital and light microscopical assessment with also a similar standard deviation; on a 4-point scale ranging from very unsure to very sure, the average score was 3.0 for conventional microscopy and 3.1 for digital pathology. The need for additional investigations was also comparable with 39% for digital assessment and 42% for light microscopical assessment.

### Experienced practical problems in digital microscopy

Practical problems were encountered in 15% of digital assessments vs. only 5% of glass assessments. These problems mostly consisted of unfocused images (73%) and insufficient speed of the system (server-dependent, 20%). Difficulty to assess morphology was noted twice (5%) and insufficient contrast once (2%).

## Discussion

Digital pathology with WSI has a large potential to make the process of expert consultation and expert panel diagnosis more rapid and more efficient. In addition to use in the consultation setting, implementation of digital pathology opens up other possibilities. It allows pathologists to work remotely, thereby allowing a more efficient use of working hours. In addition, the archive is immediately available for additional questions from clinicians or to compare with follow-up biopsies. Also, the digital availability of the images allows the application of algorithms. This has the potential for more objective and faster scoring of known diagnostic criteria (e.g., immunohistochemical stains, mitotic count), and it could also lead to the discovery of new relevant morphological features by the application of machine learning. To implement digital pathology requires significant investments in hardware, software, and IT infrastructure to ensure reliable and safe use, both within the laboratory and remotely. Also, comparison with the current methods is necessary for validation of the technique. Multiple large studies have shown the non-inferiority of digital pathology for routine diagnosis, using multiple platforms and assessing different organ systems [6, 8, 9].

In this study, we evaluated the performance of WSI versus conventional microscopy for the diagnosis of hematopathological diseases with a focus on lymphoid malignancies. We conclude that the performance of assessment by digital pathology is in general comparable with that of conventional light microscopy and that pathologists feel confident using digital pathology for this subspecialty.

Discordances between conventional microscopy and digital pathology were present in 3% of assessments. This is comparable with previous studies, which show a discordance rate of 3–4% [8, 10].

With respect to the discordances identified in this study, three out of five of these discordances consisted of a diagnosis of diffuse large B cell lymphoma on digital pathology, but an indolent B cell lymphoma by light microscopy. Therefore, overestimation of the number of blasts in a lymphoma could be a pitfall of digital hematopathology. It was noted by one of the pathologists that the nuclear details were difficult to assess with digital pathology. This could be due to the inability to adjust the focus with digital microscopy in thick slides and calls for consistent thin slides and scanning of slides at multiple levels (Z-stacking). Further studies into this subject would be interesting since the diagnosis of histological progression of indolent lymphoma is a well-known difficult area with a lack of clear-cut definitions for blast numbers and morphology. The detected discordances also seem to be a reflection of this difficulty. The rapidly expanding application of image analysis on WSI might in the future help solving this problem. The major discordances that we observed in this study are expected to have clinical implications. The most frequent type of discordance was a low-grade versus an aggressive lymphoma, which could result in another therapeutic regimen. In two other discordances with a more pronounced change in diagnosis (follicular lymphoma vs. classical Hodgkin lymphoma and T cell lymphoma vs. classical Hodgkin lymphoma), this would also probably result in another treatment. Finally, in two cases, the discordance was a diagnosis of lymphoma in a reactive condition or vice versa, which is expected to result in unnecessary treatment or a delay of the diagnosis.

At the time of the study, the pathologists involved did not yet use digital pathology for daily routine diagnostics. Yet, the level of confidence was similar for light microscopy and digital pathology. This shows that also pathologists with limited experience in digital pathology are able to join a digital hematopathology expert panel or review digital hematopathological consultation cases.

With respect to the practical assessment of digital images, important improvements can be made. These mostly consist of technical improvements in image focus and speed of the system. It can be expected that with the implementation of digital pathology in routine diagnostic practice, these issues will be resolved. In this study, we did not make a comparison of the time it took to review each case by digital or conventional microscopy. It would also have been questionable to record the time for digital microscopy in this study, because the pathologists did not use dedicated equipment for digital pathology and were therefore probably less efficient than they would have been on a dedicated work station.

In this study, there was a clear focus on the diagnosis of lymphoma and reactive conditions of the lymph nodes. Other hematological organs (e.g., bone marrow, spleen, thymus) were not included in the assessment. Also, although quite a broad range of diseases was covered, not all rare types of lymphoma were included in the study. Therefore, additional investigations into other hematological organs and specific types of hematological conditions might uncover other areas that require specific attention in the digital reporting of hematopathology. Finally, in actual diagnostic practice, the case mix will probably be enriched for more difficult cases as it is not usual to submit each lymphoma for panel consultation.
